# Cardiopulmonary Exercise Testing in Patients with Heart Failure: Impact of Gender in Predictive Value for Heart Transplantation Listing

**DOI:** 10.3390/life13101985

**Published:** 2023-09-29

**Authors:** Pedro Garcia Brás, António Valentim Gonçalves, João Ferreira Reis, Rita Ilhão Moreira, Tiago Pereira-da-Silva, Pedro Rio, Ana Teresa Timóteo, Sofia Silva, Rui M. Soares, Rui Cruz Ferreira

**Affiliations:** 1 Cardiology Department, Santa Marta Hospital, Central Lisbon Hospital University Center, 1169-024 Lisbon, Portugalruimsoares3@gmail.com (R.M.S.);; 2 NOVA Medical School, Faculdade de Ciências Médicas (NMS|FCM), 1169-056 Lisbon, Portugal

**Keywords:** gender, heart failure, heart transplantation, cardiopulmonary exercise testing, peak O_2_ consumption

## Abstract

Background: Exercise testing is key in the risk stratification of patients with heart failure (HF). There are scarce data on its prognostic power in women. Our aim was to assess the predictive value of the heart transplantation (HTx) thresholds in HF in women and in men. Methods: Prospective evaluation of HF patients who underwent cardiopulmonary exercise testing (CPET) from 2009 to 2018 for the composite endpoint of cardiovascular mortality and urgent HTx. Results: A total of 458 patients underwent CPET, with a composite endpoint frequency of 10.5% in females vs. 16.0% in males in 36-month follow-up. Peak VO_2_ (pVO_2_), VE/VCO_2_ slope and percent of predicted pVO_2_ were independent discriminators of the composite endpoint, particularly in women. The International Society for Heart Lung Transplantation recommended values of pVO_2_ ≤ 12 mL/kg/min or ≤14 if the patient is intolerant to β-blockers, VE/VCO_2_ slope > 35, and percent of predicted pVO_2_ ≤ 50% showed a higher diagnostic effectiveness in women. Specific pVO_2_, VE/VCO_2_ slope and percent of predicted pVO_2_ cut-offs in each sex group presented a higher prognostic power than the recommended thresholds. Conclusion: Individualized sex-specific thresholds may improve patient selection for HTx. More evidence is needed to address sex differences in HF risk stratification.

## 1. Introduction

Cardiopulmonary exercise testing (CPET) is a critical complementary test in the evaluation of patients with heart failure (HF) with reduced ejection fraction (HFrEF), particularly in selectin patients who may benefit from heart transplantation (HTx) [1,2]. Peak O_2_ consumption (pVO_2_) [3,4,5] and the VE/VCO_2_ slope (minute ventilation–CO_2_ production relationship) [3,5,6] are reliable indicators of heart failure events. A cut-off for pVO_2_ of ≤12 mL/kg/min is recommended to guide HTx listing for patients receiving β-blocker therapy, and a cut-off of 14 mL/kg/min may be used for patients intolerant to β-blockers, according to the 2016 International Society for Heart Lung Transplantation (ISHLT) listing criteria for HTx [7,8]. In female patients, alternative parameters such as a VE/VCO_2_ slope of >35 and a percent of predicted pVO_2_ ≤ 50% may be considered to guide HTx listing [7]. However, the data supporting these values come from studies that enrolled mostly male patients, with a sample that was between 80 and 90 percent male [2,5].

Indeed, female patients are underrepresented in HFrEF trials, although they account for around half of the adult HFrEF population [9]. Notably, in studies exploring CPET parameters in HFrEF, this gap in female representation is even larger [1,10,11,12]. Thus, the current evidence on female HFrEF pathophysiology and exercise testing prognostic power is scarce, and therapy changes, risk stratification, and recommendations for advanced HF therapies may be impacted by our insufficient comprehension of potential sex variations in HF [13,14]. Several trials evaluating pVO_2_ have reported lower values in female patients compared to male patients, which can be explained by anatomical and physiological differences [9,15]. Female patients exhibit lower left ventricular dimensions, with lower stroke volume and lower diastolic compliance [16,17]; women show a higher prevalence of iron deficiency, have lower hemoglobin levels [18], and have inferior lean mass compared to male patients [9,19].

The HF-ACTION trial [10] assessed the prognostic power of CPET variables to predict all-cause mortality in HFrEF and reported that the prognosis associated with a given pVO_2_ differed by sex. Female patients generally present a more favorable outcome, and have a lower pVO_2_ and a higher percent of predicted pVO_2_ [1,10]. Taking into account the sex-based variations in the pathophysiology and development of HFrEF, several authors proposed that prognostic values for pVO_2_ and VE/VCO_2_ slope should be tailored for different patient populations [12,14,20]. According to the ISHLT [7], different CPET variables can be used for risk stratification in women. Nevertheless, there is insufficient evidence to support these at this time, as unbiased data are not available [1].

This study’s objective was to assess the predictive power of the traditional HTx CPET cut-off values in HF patients, comparing women and men.

## 2. Materials and Methods

### 2.1. Study Population

From 2009 to 2018, we performed a retrospective study of a prospective database in our center. We assessed consecutive HfrEF patients who underwent CPET, were in New York Heart Association (NYHA) classes II or III, and presented left ventricular (LV) dysfunction (LV ejection fraction ≤ 40%). Patients were referred to the Heart Failure team for evaluation to determine whether HTx or mechanical circulatory support (MCS) were indicated.

### 2.2. Study Protocol

The patient’s comorbidities, HF etiology, medication, NYHA class, HFSS (Heart Failure Survival Score) [21], laboratory tests, CPET data, and electrocardiographic and echocardiographic results were evaluated.

### 2.3. Patients Were Excluded If One of the following Was Present

Age under 18 years; submaximal CPET (peak RER of ≤1.05 [7]); previous HTx or elective HTx during follow-up; coronary revascularization in the last six months; concomitant conditions limiting maximal exercise, including previous stroke, peripheral arterial disease, or musculoskeletal conditions.

### 2.4. Cardiorespiratory Exercise Testing

The modified Bruce protocol was employed to assess maximal exercise tolerance on a GE Marquette Series 2000 treadmill, with equipment calibration before each exercise exam. The VE, VO_2_, and VCO_2_ values were acquired with a Vmax 229 (SensorMedics, Yorba Linda, CA, USA) gas analyzer. Continuous ECG monitoring was used to assess the heart rate (HRt). Blood pressure (BP) was obtained with a sphygmomanometer, and O_2_ saturation was tracked with pulse oximetry. An exercise test was considered maximal if the RER (respiratory exchange ratio) was above 1.05 [7].

The pVO_2_ was defined as the highest achieved 30 s average in maximal exercise, which was then normalised for body mass. The standard methods (combining V-slope preferentially and ventilatory equivalents) were used to determine the gas exchange threshold (GET). The VE/VCO_2_ slope was determined with the least squares linear regression. The minimum ventilatory equivalent for oxygen (minimum VE/VO_2_) was employed to calculate the COP (cardiorespiratory optimal point). The partial pressure of end-tidal carbon dioxide (PetCO_2_) was recorded both before exercise and at GET. Peak O_2_ pulse, measured in millilitres per beat, was computed by dividing the derived pVO_2_ by the highest HRt during exercise. The peak systolic BP was divided by the VE/VCO_2_ slope to determine the ventilatory power. The circulatory power was estimated by multiplying the peak systolic BP by pVO_2_. The heart rate reserve was calculated using the difference between the highest HRt attained during maximal effort and the resting heart rate. The difference between the maximal heart rate attained with exercise and the heart rate one minute in recovery was used to determine the HRt recovery.

### 2.5. Follow-Up and Endpoint

All patients with HF were under follow-up for a 36-month period. The composite endpoint was defined as the combination of cardiovascular mortality or urgent HTx. Data were collected from medical records from inpatient and outpatient visits.

### 2.6. Statistical Analysis

All analytical tests compared patients according to female or male sex. Statistical analyses were performed with the Statistical Package for the Social Sciences (SPSS) v23.0.

Regarding categorical variables, results were reported as absolute frequency (number) and relative frequency (%). Continuous variables were presented as mean and standard deviation if normal distribution, or as median and interquartile range (IQR) if non-normal distribution. Normality assumptions were tested using the Kolmogorov–Smirnov test and a visual histogram analysis.

The comparison of categorical variables was performed using the Pearson’s *X*^2^ test. The Mann–Whitney U test was used to compare variables with non-normal distribution and the Student’s *t*-test was used to compare variables with normal distribution.

The correlation between the CPET parameters and the composite endpoint was evaluated using a Cox hazards regression analysis. Variables presenting a *p*-value < 0.200 in the univariate analysis were included in a multivariate analysis, adjusted for potential confounders, in order to identify independent predictors of the composite endpoint and calculate adjusted hazard ratios (HR) in each sex subgroup. The HR and the 95% confidence interval (CI) were used to report the results.

A receiver operating characteristic (ROC) curve analysis was used to examine the sensitivity and specificity of each CPET parameter in predicting the composite endpoint, in accordance with the thresholds defined by the ISHLT [7]: pVO_2_ ≤ 12 mL/Kg/min (pVO_2_ ≤ 14 in patients not tolerant to β-blockers), VE/VCO_2_ slope > 35 and percent of predicted pVO_2_ ≤ 50%.

The threshold with the highest combination of specificity and sensitivity was estimated using the Youden index (*J*). The DeLong test [22] was employed to evaluate the difference in area under the curve (AUC) between groups. Additionally, the Kaplan–Meier analysis was used to assess the event-free survival rate. A log-rank test was performed to compare the sex subgroups based on the different pVO_2_, VE/VCO_2_ slope, and percent of predicted pVO_2_ thresholds indicated by the ISHLT [7] and based on the proposed cut-offs. A significance threshold of α = 5% was considered whenever a statistical hypothesis was being tested.

## 3. Results

### 3.1. Patient Characteristics

**Figure 1:**
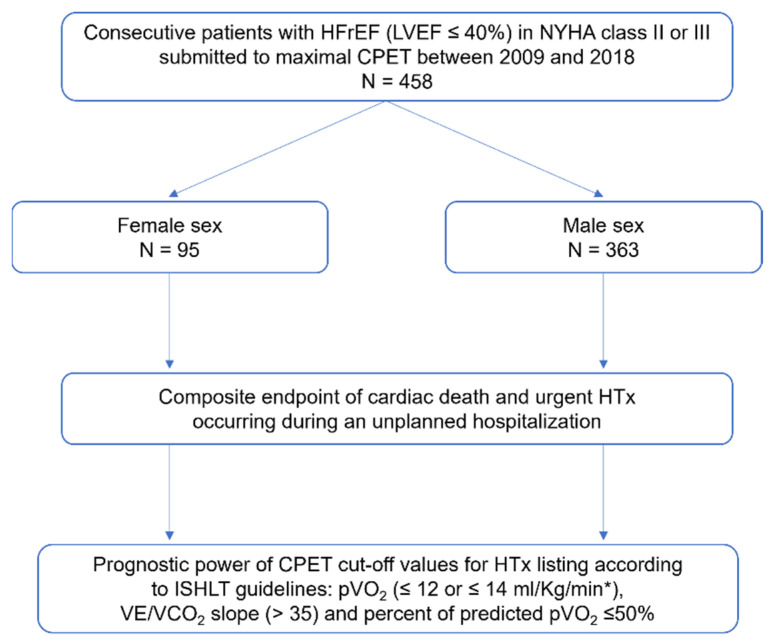
Flowchart of the study population. * in patients intolerant to β-blockers. HFrEF: Heart failure with reduced ejection fraction; LVEF: Left ventricular ejection fraction; NYHA: New York Heart Association; CPET: Cardiopulmonary exercise test; HTx: Heart transplantation; ISHLT: International Society for Heart and Lung Transplantation; pVO_2_: Peak oxygen consumption; VE/VCO_2_ slope: Minute ventilation–carbon dioxide production relationship.

Our study included 458 patients who underwent maximal exercise testing (Figure 1). Of these patients, 79% were men, 57% had ischemic etiology, 76% were in NYHA II and 24% in NYHA III, with a mean LVEF of 29.7 ± 8.0%, and 24% had atrial fibrillation (AF). In addition, 79% were taking either an ACEi (angiotensin-converting enzyme inhibitor) or an ARB (angiotensin receptor blockers), with 17% on an angiotensin receptor/neprilysin inhibitor. Mineralocorticoid receptor antagonists (MRAs) were being taken by 73% and β-blockers by 86%. Additionally, sodium-glucose cotransporter-2 inhibitors (SGLT2i) were being taken by 10% of the patients; 64% of patients had an ICD and 22% had a cardiac resynchronization device (CRT-D). Moreover, there was no difference in the mean Heart Failure Survival Score (HFSS). Compared to male patients, female patients had a similar pVO_2_ and a higher percent of predicted pVO_2_. The mean respiratory exchange ratio (RER) was 1.14 ± 0.07. Table 1 lists the baseline characteristics of both groups as well as the CPET values.

### 3.2. Composite Endpoint

The composite endpoint occurred in 68 (14.8%) patients in 36 months of follow-up, with cardiovascular death occurring in 54 individuals and urgent HTx occurring in 14 patients (Table 2). No urgent MCS was required; 10.5% of female patients and 16.0% of male patients experienced the composite endpoint, with no significant difference between groups.

### 3.3. Prognostic Power of CPET Parameters

The pVO_2_ (HR 0.856), the VE/VCO_2_ slope (HR 1.064), and the percent of predicted pVO_2_ (HR 0.955) were associated with the composite endpoint in a multivariable Cox regression analysis, regardless of the sex subgroup. Table 3 displays the results of the uni- and multivariable models. The correlations in the multivariable model were independent of potential confounders such as body mass index, LVEF, age, sex, smoking, diabetes mellitus, or estimated glomerular filtration rate. In the multivariable analysis, most of the other exercise testing variables were not linked with the primary endpoint. The peak O_2_ pulse was associated with the endpoint in both female and male patients. The ventilatory power, the circulatory power, and the PetCO_2_ at GET were linked with the primary endpoint in male patients, as shown in Table 3.

**Figure 2:**
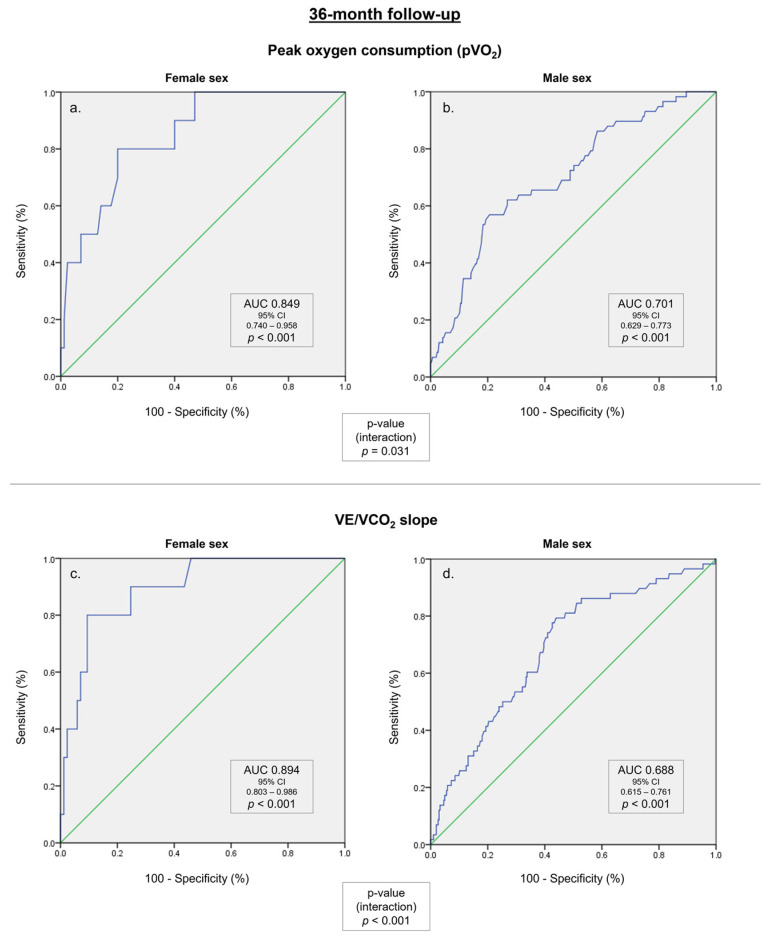
ROC curves for the composite endpoint in a 36-month follow up. (**a**) Peak oxygen consumption (pVO_2_) in female patients. (**b**) pVO_2_ in male patients. (**c**) Minute ventilation–carbon dioxide production relationship (VE/VCO_2_ slope) in female patients. (**d**) VE/VCO_2_ slope in male patients.

In an ROC curve analysis, the pVO_2_, the VE/VCO_2_ slope, and the percent of predicted pVO_2_ were linked to the composite endpoint, both in females and males. The predictive ability of these variables was significantly higher in women compared to males, including for pVO_2_, VE/VCO_2_ slope, and the percent of predicted pVO_2_, as presented in Table 4. The ROC curves for these subgroups are illustrated in Figure 2 and Supplementary Figure S1. In addition, the predictive power of the peak O_2_ pulse was also significantly higher in female patients compared to males (AUC 0.816 vs. AUC 0.616, *p* = 0.023).

The circulatory power presented a slightly higher prognostic power than the recommended exercise testing parameters in men (AUC 0.713 vs. AUC 0.701, *p* = 0.161), albeit with no statistically significant differences in predictive power. Despite being significant predictors of the composite endpoint, additional CPET variables such as peak O_2_ pulse, ventilatory power, COP, PetCO_2_ at rest, and PetCO_2_ at GET had an inferior predictive power than the traditional CPET parameters (Table 4).

### 3.4. ISHLT Recommended Thresholds for HTx Listing

**Figure 3:**
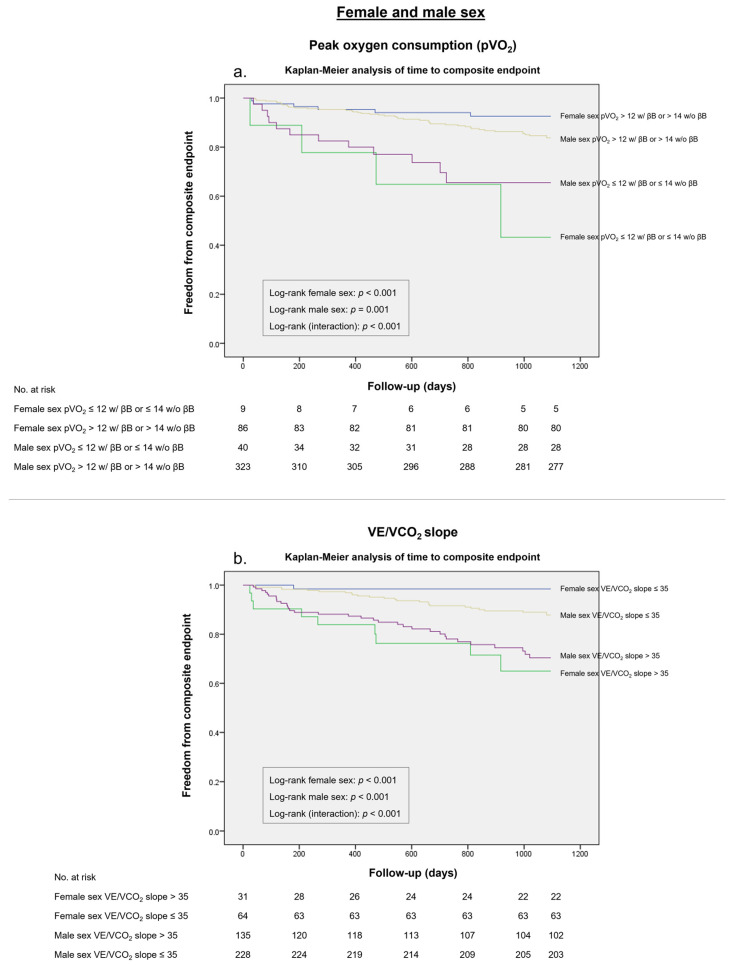
Survival analysis for the composite endpoint according to the International Society for Heart and Lung Transplantation (ISHLT) thresholds in female patients and male patients. (**a**) Peak oxygen consumption (pVO_2_) ≤ 12 mL/Kg/min (≤14 mL/kg/min if intolerant to β-blockers [βB]). (**b**) Minute ventilation–carbon dioxide production ratio (VE/VCO_2_ slope) of >35.

A pVO_2_ of ≤ 12 mL/kg/min (≤14 if the patient is intolerant to β-blockers) was present in 49 (11%) patients. This threshold was linked with poor HF outcomes (HR 3.487, *p* < 0.001). This pVO_2_ cut-off showed a sensitivity of 40% and a specificity of 94% in women, presenting a higher Youden index compared to men (*J* 0.34 vs. *J* 0.12), with a sensitivity of 21% and a specificity of 91%, as shown in Table 5. This cut-off was shown to be a strong discriminator of HF outcomes for both sex subgroups in a Kaplan–Meier analysis (Figure 3a).

A total of 166 (36%) patients showed a VE/VCO_2_ slope value higher than 35. The composite endpoint occurred at a higher rate in individuals over this threshold as well (HR 3.587, 95% CI 2.194–5.864, *p* < 0.001). This threshold revealed a substantially higher Youden index in women (*J* 0.65 vs. *J* 0.23), with sensitivity of 90% and a specificity of 75%, in comparison with male patients, with a sensitivity of 57% and a specificity of 66%. In the survival analysis, this VE/VCO_2_ slope cut-off was a reliable indicator of the composite endpoint in both sex categories (Figure 3b).

In our cohort, a percent of predicted pVO_2_ of less than 50% was present in 120 (26%) patients. This cut-off was associated with the composite endpoint (HR 4.355, 95% CI 2.694–7.039, *p* < 0.001). This cut-off showed a sensitivity of 60% and a specificity of 89% in females, while it had a sensitivity of 48% and specificity of 78% in males. As a result, the Youden index in females was higher than in male patients (*J* 0.49 vs. *J* 0.26). This threshold was a reliable discriminator in both subgroups according to the survival curve analysis (Supplementary Figure S2a).

### 3.5. Alternative Thresholds for pVO_2_ and VE/VCO_2_ Slope

**Figure 4:**
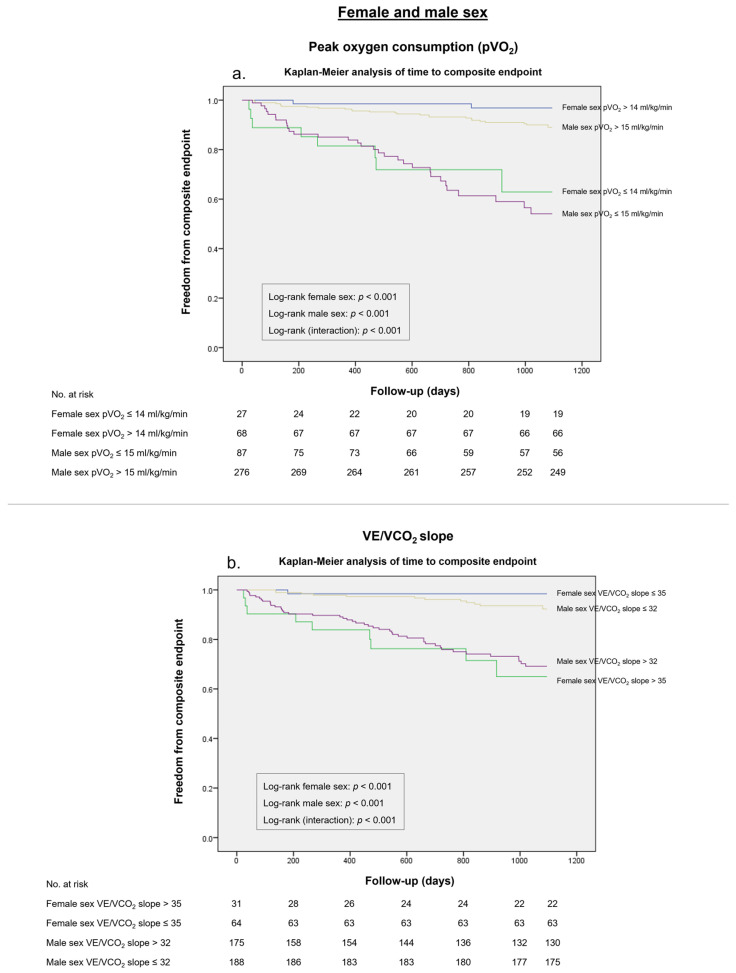
Survival analysis for the composite endpoint in female patients and male patients according to (**a**) Peak O_2_ consumption (pVO_2_) of ≤ 14 and ≤ 15 mL/Kg/min, respectively. (**b**) Minute ventilation–CO_2_ production ratio (VE/VCO_2_ slope) of > 35 and > 32, respectively.

In an assessment of potential alternative thresholds, a pVO_2_ ≤ 14 mL/kg/min yielded a higher Youden index in female patients compared to the pVO_2_ ≤ 12 mL/kg/min cut-off (*J* 0.60 vs. *J* 0.34) (Table 5). Similarly, a pVO_2_ ≤ 15 mL/kg/min value showed a higher overall diagnostic effectiveness in male patients compared to the traditional cut-off (*J* 0.36 vs. *J* 0.12). The predictive value of this cut-off was supported by the Kaplan–Meier analysis (Figure 4a).

In males, a VE/VCO_2_ slope threshold of > 32 demonstrated sensitivity of 78% and a specificity of 57%, exhibiting a higher Youden index than the traditional VE/VCO_2_ slope cut-off (*J* 0.35 vs. *J* 0.23). Regarding female patients, the traditional VE/VCO_2_ slope > 35 threshold was associated with the highest overall diagnostic effectiveness (*J* 0.65). Additionally, it was demonstrated in the survival analysis that these cut-off values accurately predicted worse outcomes (Figure 4b).

A percent of predicted pVO_2_ of ≤ 55% yielded a significantly higher Youden index in female patients compared to the ≤ 50% threshold *(J* 0.76 vs. *J* 0.49) while a percent of predicted pVO_2_ of ≤ 58% showed a higher Youden index in comparison to the traditional thresholds (*J* 0.32 vs. *J* 0.26) (Table 5). These cut-offs were accurate discriminators of the composite endpoint in both sex subgroups (log-rank *p* < 0.001) (Supplementary Figure S2b).

## 4. Discussion

Our study’s key conclusion was that the traditional CPET variables had a considerably higher predictive power for HF outcomes in women compared to men. Furthermore, the ISHLT recommended thresholds for pVO_2_ (≤12 mL/kg/min, or ≤ 14 mL/kg/min if intolerant to β-blockers), VE/VCO_2_ slope (>35), and percent of predicted pVO_2_ ≤ 55% showed a significantly higher overall diagnostic effectiveness in women compared to men. Additionally, our study assessed the predictive capacity of various CPET variables and proposed sex-specific cut-offs for pVO_2_, VE/VCO_2_ slope, and the percent of predicted pVO_2_, which may assist in a more precise risk assessment in women and men with HFrEF. However, one of the main limitations of our study was that 79% of the enrolled patients were male; thus, further studies should include a higher proportion of female patients.

The current evidence on the predictive value of CPET in women with HFrEF was evaluated in a recent article by the Heart Failure Association’s Committee on Exercise Physiology and Training [1,14,23,24,25,26,27]. The mean age of female patients enrolled in these studies was slightly lower than that of male patients, and one of the explanations for female underrepresentation in HfrEF trials was a larger proportion of older women who were excluded due to the policy of non-inclusion of elderly patients [28]. 

pVO_2_ is influenced by gender, age, motivation, pulmonary status, and muscle mass [29], which raised concerns that this parameter’s role as a prognostic indicator in female patients may lead to premature cardiac transplantation in women [14].

However, several observational studies showed that pVO_2_ is a reliable discriminator for HF events in female patients [24,26] and a large trial [12] showed that predictive pVO_2_ cut-offs for men and women with HfrEF should be independent. Although thresholds such as the GET were described to provide incremental value in the assessment of cardiorespiratory fitness in healthy controls [30,31], VO_2_ measured at GET did not show a significant prognostic power compared to pVO_2_ in our HfrEF cohort.

Women generally exhibited a lower corrected pVO_2_ than male patients; however, female patients presented a lower rate of HF events. Notably, female patients showed a nearly 10% higher percent of predicted pVO_2_ compared to men [23,27]. However, a study by Corrà et al. [11] postulated that HF outcomes in women may not actually be better than in men, as the female prognostic advantage is lost when sex-specific variations are properly taken into account with propensity score matching. Therefore, adjusting for sex-related characteristics should be undertaken. Indeed, female patients in our cohort showed a significantly higher percent of predicted pVO_2_ despite having a similar absolute pVO_2_ value, with a numerically inferior frequency of the composite endpoint. 

The VE/VCO_2_ slope is an alternative CPET parameter with proven prognostic power, and the HF event risk is constant throughout a large range of VE/VCO_2_ slope values [32,33,34]. A study by Guazzi et al. [25] demonstrated that in both men and women with HfrEF, the predictive power of pVO_2_ and the VE/VCO_2_ slope are similar. Notably, the discriminative power of the VE/VCO_2_ slope was greater than that of pVO_2_ in female patients. Our findings are in keeping with these results, as the VE/VCO_2_ slope also showed a slightly higher prognostic power compared to pVO_2_ in the ROC curve analysis in the female subgroup.

The percent of predicted pVO_2_, an age- and gender-adjusted parameter assessing exercise capacity, was shown to stratify the risk for HF events with a higher accuracy compared to pVO_2_ in women [27]. The role of CPET in pre-surgical risk stratification in women has also been studied. In a study by Rose et al. [35,36], sex-specific CPET thresholds improved surgical risk stratification and thus may contribute to optimise clinical decision-making.

There is a paucity of randomized clinical trials evaluating the value of CPET variables in women with HFrEF. The HF-ACTION [10], a randomized trial with 2100 patients, also concluded that women presented a better clinical outcome, showing a lower pVO_2_ and a higher percent of predicted pVO_2_ compared to men. The parameter with the highest predictive power in women was the percent of predicted pVO_2_. Our study had similar findings, as the percent of predicted pVO_2_ was the CPET parameter with the highest predictive power for HF outcomes in the female subgroup. This result is in keeping with the ISHLT guidelines [7], which recommend that alternative parameters such as percent of predicted pVO_2_ may be considered in conjunction with pVO_2_ to guide HTx listing in female patients. In contrast, in the male subgroup, pVO_2_ and percent of predicted pVO_2_ had a similar prognostic power for risk stratification of HF events. Moreover, our study showed that, in a cohort with similar pVO_2_ values between sexes, the predictive power of the traditional CPET parameters was notably lower in men than in women, which is in contrast with the results reported in a previous trial by Elmariah et al. [14].

The position paper by Corrà et al. [1] proposes three different threshold values of pVO_2_ for male HFrEF patients: <10 mL/kg/min, 10 to 18, or > 18 mL/kg/min. However, there are still limited data to define an accurate cut-off for other subgroups of patients, women or elderly patients in particular [9]. Extrapolating these three advocated thresholds of pVO_2_ in male patients to other subgroups may lead to misconceptions and inaccuracies of the objective pVO_2_ [9]. Consequently, further studies are necessary to define an accurate threshold to guide patient selection for HTx listing in women. 

In a trial by Green et al. [26], the proposed pVO_2_ thresholds in females with HFrEF for high- (≤10), medium- (10.1 to 14), and low-risk (>14 mL/kg/min) showed a one-year event-free survival of 80%, 84%, and 93%, respectively. Elmariah et al. [14] reported that, in the current era, HTx may be deferred if the pVO_2_ is over 10 mL/kg/min. However, this study had several disparities between sexes in the baseline characteristics and it did not consider patients with CRT, which can affect pVO_2_ values [37,38].

In our cohort, the ISHLT recommended thresholds of pVO_2_ ≤ 12 or ≤ 14 mL/kg/min, VE/VCO_2_ slope > 35 and percent of predicted pVO_2_ ≤ 50% showed a higher overall diagnostic effectiveness in women compared to men, in keeping with the higher prognostic power these parameters showed in female patients. We proposed alternative thresholds that may improve risk discrimination among female patients. A threshold of pVO_2_ ≤ 14 mL/kg/min (including patients on β-blockers) and a percent of predicted pVO_2_ ≤ 55% showed a slightly lower specificity but a higher sensitivity, with an overall higher overall diagnostic effectiveness. The recommended cut-off of VE/VCO_2_ slope > 35 was the strongest predictor of HF events in women. Regarding male patients, a pVO_2_ threshold of ≤ 15 mL/kg/min, a VE/VCO_2_ slope of > 32, and a percent of predicted pVO_2_ of ≤ 58% may also provide an improved diagnostic effectiveness compared to the traditional thresholds for HTx listing.

Circulatory power is a surrogate of left ventricular stroke work index, incorporating pVO_2_ and peak systolic BP [39]. Circulatory power was a significant predictor of HF events in our cohort, especially in males. In a recent study by Martinez et al. [40] evaluating patients with advanced HF, circulatory power presented the highest discriminative power for HF outcomes and mortality, concluding that this parameter should also be considered for risk stratification in conjunction with the traditional CPET variables. However, further research is needed to determine whether circulatory power can contribute to the decision of the optimal timing for HTx in women.

In our study, peak O_2_ pulse presented a significantly higher predictive power for HF outcomes in women. Peak O_2_ pulse, a non-invasive measure of stroke volume and arteriovenous O_2_ differential, represents the pVO_2_ corrected for HRt [41]. Several CPET measures, including the pVO_2_, are corrected for total weight rather than lean body mass. There is a high variability in body fat as a percentage of total body weight [27,42,43] which can also contribute to the lower pVO_2_ reported in women [44]. The use of corrected pVO_2_ adjusted for lean body mass may be a more accurate measurement of exercise intolerance, particularly in groups with a greater body fat percentage such as women [42,45].

A trial by Lavie [45] et al. found that pVO_2_ lean and peak O_2_ pulse lean outperformed pVO_2_ as predictors of major HF events, including among obese patients and women. The authors noted that, when combined with conventional CPET variables, peak O_2_ pulse and lean body mass-adjusted O_2_ pulse were powerful predictors of HF outcomes in patients with HFrEF, particularly in populations with a higher percent of body fat.

Prognostic risk scores such as a high to medium risk HFSS [21] or a Seattle Heart Failure Model (SHFM) [46,47] <80% are also recommended as alternative parameters to consider HTx listing [7]. Although the SHFM was also an accurate predictor of HF outcomes in female patients, the Meta-Analysis Global Group in Chronic Heart Failure (MAGGIC) [48,49] score outperformed the SHFM owing to improved risk classification, presenting a similar discriminatory ability in both sexes, despite an overestimation of death in female patients at the 3-year follow-up [50].

### Limitations

Firstly, since this is a retrospective study of a prospective database in our center, our findings require confirmation in larger, randomized studies. Additionally, the majority of patients enrolled were men (79%), which is a high proportion, particularly in a study evaluating disparities between female and male patients.

Secondly, our study enrolled unmatched patient subgroups. However, consecutive patient enrollment attenuated the lack of randomization. Furthermore, most baseline characteristics were comparable between sex groups. Men had a numerically higher proportion of ischemic HF, although there was no statistical difference among groups.

Most patients in each subgroup were receiving optimal disease-modifying pharmacological therapies for HF. However, only 10% of patients in our cohort were taking SGLT2i, as they were included in our study between 2009 and 2018, when this drug class was not yet considered as an optimised standard of care medication for patients without diabetes [51]. Future studies should include more patients taking SGLT2i, as they have shown to significantly reduce HF events [52,53]. Less than 25% of patients were taking angiotensin receptor/neprilysin inhibitors, as this therapy was not available for patients enrolled before 2016. Future trials should include more patients receiving sacubitril/valsartan. Moreover, new therapies such as selective cardiac myosin activators or guanylate cyclase stimulators were not available at the time of patient enrolment.

Thirdly, our research lacked the statistical power to infer a new pVO_2_ threshold for patients who were intolerant to β-blockers, as the majority of patients (86%) were taking β-blockers. As a result, the proposed pVO_2_ thresholds might not be reproducible in this subgroup of HFrEF patients.

Our study only included patients who had a maximal CPET. There is no current agreement on the best peak RER cut-off to determine maximal effort, especially in patients with HFrEF. A number of cut-offs ranging from 1.0 to 1.10 were suggested [1,54,55,56]. As our aim was to assess the recommendations for HTx, a peak RER of 1.05 was considered to determine a maximal CPET, as recommended by the ISHLT [7]. Consequently, our proposed cut-offs might not be applicable to an HF population with submaximal exercise testing, particularly considering the lower prognostic power of pVO_2_ in submaximal exercise capacity [57]. In patients with submaximal exercise capacity, VE/VCO_2_ slope and percent of predicted pVO_2_ may assist in the clinical stratification [7,34,55]. Indeed, the reliability of RER-based assessment of maximal exercise is suboptimal as there are methodological issues thwarting the accurate assessment of VO_2_max in submaximal exercise. Pool and Jones [58] caution against the acceptance of pVO_2_ measured during ramp incremental exercise as a maximum value in patients with submaximal exercise and proposed the inclusion of a second short constant work rate CPET, completed at a higher work rate than that previously achieved during the ramp test, in order to accurately verify the VO_2_max. Therefore, serial CPET may be more informative than a single cardiopulmonary exercise test and thus provide a more accurate assessment of the VO_2_max.

Our study evaluated the GET, as described by Beaver et al. [59]. However, it is now recognized that insufficient O_2_ is not the primary basis for lactatemia. Critical power likely represents the threshold above which there is a sustained glycolytic contribution with lactate accumulation. Although lactate is a key energy source, there is no evidence that the muscle becomes dysoxic or anoxic [60]. Thus, instead of the GET, critical power may potentially be a more accurate predictor of exercise capacity [60].

Lastly, our study had a lower rate of HF outcomes, especially urgent HTx, compared to other studies [32]. As all the recruited patients were reviewed by the specialized Heart Failure team for a possible indication for HTx, our results may not be applicable to the overall HFrEF population encompassing older patients or patients with significant comorbidities.

## 5. Conclusions

In an HFrEF cohort undergoing CPET, pVO_2_, VE/VCO_2_ slope, and the percent of predicted pVO_2_ were the variables with the highest discriminative power for HF events, with a higher predictive power in female patients compared to male patients. The ISHLT guideline thresholds for pVO_2_ and VE/VCO_2_ slope showed a higher diagnostic effectiveness in women. Sex-specific pVO_2_, VE/VCO_2_ slope, and percent of predicted pVO_2_ cut-offs presented a higher prognostic power than the recommended thresholds. Our results indicate that sex-specific cut-offs may assist in patient selection for HTx. However, more data are necessary to help close the gap in evidence between sexes.

## Figures and Tables

**Table 1:** Baseline characteristics of the study population (*n* = 458).

	Overall (*n* = 458)	Female (*n* = 95)	Male (*n* = 363)	*p*-Value
Clinical and demographic data				
Age (years)	56 ± 12	54 ± 14	56 ± 12	0.328
Body mass index (kg/m^2^)	27.1 ± 4.3	26.3 ± 4.6	27.4 ± 4.2	0.335
Ischemic etiology (*n*, %)	261 (57)	47 (49)	214 (59)	0.092
ACEi/ARB (*n*, %)	361 (79)	77 (81)	284 (78)	0.199
ARNI (*n*, %)	80 (17)	13 (14)	67 (18)	0.273
β-blocker (*n*, %)	392 (86)	81 (85)	311 (86)	0.726
MRA (*n*, %)	336 (73)	72 (76)	264 (73)	0.789
iSGLT2 (*n*, %)	47 (10)	8 (8)	39 (11)	0.164
Digoxin (*n*, %)	129 (28)	23 (24)	106 (29)	0.372
Diabetes	104 (23)	15 (16)	89 (25)	0.094
CKD (*n*, %)	145 (32)	25 (26)	120 (34)	0.138
AF (*n*, %)	109 (24)	14 (15)	95 (26)	0.021
ICD * (*n*, %)	293 (64)	59 (62)	234 (64)	0.617
Cardiac resynchronization therapy (*n*, %)	102 (22)	27 (28)	75 (21)	0.128
NYHA II	347 (76)	74 (78)	273 (75)	0.485
NYHA III	111 (24)	21 (22)	90 (25)	0.485
HFSS	8.6 ± 1.1	8.8 ± 0.9	8.6 ± 1.2	0.109
Laboratory data				
eGFR, ml/min/1.73 m^2^	75.3 ± 29.2	77.1 ± 30.9	74.8 ± 28.7	0.517
Na^+^, mEq/l	138.0 ± 3.0	138.4 ± 2.8	137.9 ± 3.1	0.108
N-terminal pro b-type natriuretic peptide, pg/mL	2196 ± 2101	2204 ± 1724	2193 ± 2099	0.979
Echocardiographic data				
LVEDD, mm/m^2^	67.4 ± 10.3	63.8 ± 9.7	68.0 ± 10.3	0.064
LVEF, %	29.7 ± 8.0	31.3 ± 7.9	29.0 ± 7.5	0.213
Mitral regurgitation severity III–IV, %	67 (14)	19 (20)	48 (13)	0.097
Right ventricular dysfunction (*n*, %)	69 (15)	9 (10)	60 (16)	0.630
Exercise testing data				
Peak Respiratory Exchange Ratio	1.14 ± 0.07	1.13 ± 0.08	1.14 ± 0.07	0.566
Delta heart rate during exercise	51 (37–68)	48 (34–67)	52 (38–69)	0.819
HHR1	17 (11–27)	19 (14–29)	16 (11–26)	0.058
pVO_2_, mL/kg/min	18.5 ± 5.8	18.0 ± 5.6	18.6 ± 5.9	0.363
Percent of predicted pVO_2_ (%)	63.8 ± 18.7	67.4 ± 16.7	62.8 ± 19.1	0.021
VE/VCO_2_ slope	33.9 ± 9.6	33.0 ± 8.9	34.2 ± 9.8	0.246
pVO_2_, mL/kg/min at GET	13.6 ± 4.6	10.9 ± 2.8	14.2 ± 4.7	0.001
Peak O_2_ pulse	0.14 ± 0.06	0.13 ± 0.03	0.14 ± 0.07	0.149
Circulatory power	2883 ± 1543	2715 ± 1035	2927 ± 1649	0.235
Ventilatory power	4.8 ± 1.7	4.8 ± 1.5	4.8 ± 1.7	0.739
COP	28.9 ± 7.2	29.5 ± 7.9	28.8 ± 7.0	0.630
PetCO_2_ at rest, mmHg	33.6 ± 4.8	33.9 ± 5.1	33.5 ± 4.7	0.558
PetCO_2_ at GET, mmHg	36.8 ± 6.0	37.5 ± 5.9	36.6 ± 6.1	0.262

* including patients with a cardiac resynchronization therapy device. CPET: Cardiopulmonary exercise test; ACEi: Angiotensin-converting enzyme inhibitors; ARNI: Angiotensin receptor neprilysin inhibitors; ARB: Angiotensin receptor blockers; MRA: Mineralocorticoid receptor antagonists; CKD: Chronic kidney disease; AF: Atrial fibrillation; ACEi: Angiotensin-converting enzyme inhibitors; ICD: Implantable cardioverter-defibrillator; HFSS: Heart Failure Survival Score; eGFR: estimated glomerular filtration rate; LVEF: Left ventricular ejection fraction; LVEDD: Left ventricular end-diastolic diameter; pVO_2_: Peak O_2_ consumption; VE/VCO_2_ slope: Minute ventilation-carbon dioxide production relationship; GET: Gas exchange threshold; COP: Cardiorespiratory optimal point; HRR1: Heart rate recovery in the first minute after finishing CPET; PetCO_2_: Partial pressure of end-tidal carbon dioxide.

**Table 2:** Total adverse events during follow-up.

	Total Cohort (*n* = 458)	Female (*n* = 95)	Male (*n* = 363)	*p*-Value
Composite endpoint (*n*, %)	68 (14.8%)	10 (10.5%)	58 (16.0%)	0.199
Total mortality (*n*, %)	67 (14.6%)	13 (13.7%)	54 (14.9%)	0.597
Cardiac mortality (*n*, %)	54 (11.8%)	8 (8.4%)	46 (12.7%)	0.098
Sudden cardiac death (*n*, %)	19 (4.1%)	2 (2.1%)	17 (4.7%)	0.147
Death from worsening HF (*n*, %)	35 (7.6%)	6 (6.3%)	29 (7.9%)	0.638
Urgent HTx (*n*, %)	14 (3.1%)	2 (2.1%)	12 (3.3%)	0.744

HF: Heart failure; HTx: Heart transplantation.

**Table 3:** Univariable and multivariable analysis of the composite endpoint.

**Total Cohort**						
Model	Univariable HR	95% CI	*p*-value	Multivariable HR	95% CI	*p*-value
Male sex	1.547	0.791–3.026	0.203			
Age	1.002	0.983–1.021	0.829			
BMI	0.953	0.897–1.013	0.121	0.954	0.887–1.027	0.210
LVEF	0.927	0.900–0.955	<0.001	0.935	0.905–0.966	<0.001
eGFR	0.979	0.969–0.989	<0.001	0.986	0.976–0.996	0.009
Diabetes	1.196	0.254–5.632	0.821			
Smoker	1.716	1.405–2.820	0.033	1.395	0.835–2.328	0.203
Peak VO_2_	0.835	0.789–0.883	<0.001	0.856	0.804–0.912	<0.001
Percent of predicted pVO_2_	0.948	0.934–0.963	<0.001	0.955	0.939–0.971	<0.001
VE/VCO_2_ slope	1.058	1.041–1.075	<0.001	1.064	1.039–1.090	<0.001
Peak VO_2_ at GET, mL/kg/min	0.854	0.737–0.989	0.035	0.879	0.687–1.124	0.305
O_2_ pulse, mL/kg/beat	0.858	0.791–0.932	<0.001	0.865	0.780–0.961	0.007
Circulatory power, mmHg.mL/kg/min	0.999	0.999–0.999	<0.001	0.999	0.998–1.000	<0.001
Ventilatory power, mmHg	0.575	0.483–0.684	<0.001	0.632	0.521–0.768	<0.001
COP	1.118	1.054–1.186	<0.001	1.060	0.956–1.174	0.268
PetCO_2_ at rest, mmHg	0.887	0.839–0.937	<0.001	0.948	0.889–1.011	0.102
PetCO_2_ at GET, mmHg	0.862	0.826–0.900	<0.001	0.890	0.845–0.993	<0.001
**Female sex**						
Model	Univariable HR	95% CI	*p*-value	Multivariable HR	95% CI	*p*-value
Age	1.003	0.960–1.048	0.888			
BMI	0.897	0.770–1.045	0.162	0.861	0.694–1.067	0.171
LVEF	0.893	0.820–0.973	0.010	0.941	0.864–1.016	0.160
eGFR	0.977	0.952–1.003	0.086	0.991	0.966–1.016	0.459
Diabetes	1.135	0.629–2.053	0.674			
Smoker	0.940	0.199–4.436	0.937	1.565	0.178–13.699	0.686
Peak VO_2_	0.704	0.583–0.850	<0.001	0.746	0.604–0.922	0.007
Percent of predicted pVO_2_	0.911	0.875–0.948	<0.001	0.913	0.858–0.972	0.004
VE/VCO_2_ slope	1.093	1.052–1.135	<0.001	1.143	1.039–1.257	0.006
Peak VO_2_ at GET, mL/kg/min	0.223	0.010–5.159	0.350			
O_2_ pulse, mL/kg/beat	0.493	0.346–0.703	<0.001	0.458	0.261–0.802	0.006
Circulatory power, mmHg.mL/kg/min	0.998	0.997–0.999	0.002	0.999	0.998–1.000	0.069
Ventilatory power, mmHg	0.405	0.240–0.684	0.001	0.565	0.297–1.072	0.080
COP	1.775	0.100–3.450	0.903			
PetCO_2_ at rest, mmHg	0.903	0.792–1.028	0.123	0.981	0.841–1.144	0.807
PetCO_2_ at GET, mmHg	0.814	0.715–0.927	0.002	0.871	0.736 –1.031	0.108
**Male sex**						
Model	Univariable HR	95% CI	*p*-value	Multivariable HR	95% CI	*p*-value
Age	1.001	0.979–1.022	0.963			
BMI	0.960	0.898–1.027	0.240			
LVEF	0.933	0.905–0.963	<0.001	0.938	0.905–0.971	<0.001
eGFR	0.980	0.969–0.991	<0.001	0.987	0.976–0.998	0.020
Diabetes	1.211	0.639–2.230	0.558			
Smoker	1.791	1.024–3.133	0.041	1.425	0.805–2.521	0.224
Peak VO_2_	0.854	0.806–0.905	<0.001	0.869	0.813–0.928	<0.001
Percent of predicted pVO_2_	0.956	0.941–0.971	<0.001	0.960	0.943–0.977	<0.001
VE/VCO_2_ slope	1.051	1.032–1.070	<0.001	1.056	1.030–1.084	<0.001
Peak VO_2_ at GET, mL/kg/min	0.862	0.746–0.996	0.044	0.880	0.691–1.121	0.302
O_2_ pulse, mL/kg/beat	0.873	0.802–0.949	0.001	0.884	0.794–0.985	0.026
Circulatory power, mmHg.mL/kg/min	0.999	0.999–0.999	<0.001	0.999	0.999–1.000	<0.001
Ventilatory power, mmHg	0.611	0.510–0.733	<0.001	0.645	0.526–0.792	<0.001
COP	1.095	1.027–1.167	0.005	1.062	0.962–1.173	0.230
PetCO_2_ at rest, mmHg	0.886	0.834–0.942	<0.001	0.937	0.873–1.005	0.070
PetCO_2_ at GET, mmHg	0.870	0.831–0.911	<0.001	0.887	0.839–0.939	<0.001

BMI: Body mass index; eGFR: Estimated glomerular filtration rate; LVEF: Left ventricular ejection fraction; pVO_2_: Peak oxygen consumption; VE/VCO_2_ slope: Minute ventilation–carbon dioxide production relationship; GET: Gas exchange threshold; COP: Cardiorespiratory optimal point; PetCO_2_: Partial pressure of end-tidal carbon dioxide.

**Table 4:** Receiver operating characteristic (ROC) curve analysis of the composite endpoint.

	Female (*n* = 95)			Male (*n* = 363)			
CPET Parameters	AUC	95% CI	*p*-Value	AUC	95% CI	*p*-Value	*p*-Value (Interaction)
pVO_2_, mL/kg/min	0.849	0.740–0.958	<0.001	0.701	0.629–0.773	<0.001	0.031
Predicted pVO_2_ (%)	0.918	0.860–0.975	<0.001	0.701	0.628–0.774	<0.001	<0.001
VE/VCO_2_ slope	0.894	0.803–0.986	<0.001	0.688	0.615–0.761	<0.001	<0.001
pVO_2_, mL/kg/min at GET	0.648	0.464–0.832	0.096	0.635	0.451–0.820	0.140	0.594
O_2_ pulse, mL/kg/beat	0.816	0.669–0.962	0.001	0.616	0.537–0.695	0.005	0.023
Circulatory power, mmHg.ml/kg/min	0.788	0.642–0.935	0.003	0.713	0.646–0.780	<0.001	0.444
Ventilatory power, mmHg	0.782	0.597–0.967	0.004	0.711	0.641–0.780	<0.001	0.504
COP	0.626	0.482–0.770	0.095	0.704	0.560–0.848	0.019	0.372
PetCO_2_ at rest, mmHg	0.606	0.390–0.822	0.275	0.654	0.580–0.728	<0.001	0.694
PetCO_2_ at GET, mmHg	0.784	0.638–0.930	0.004	0.719	0.644–0.794	<0.001	0.461

CPET: Cardiopulmonary exercise testing; pVO_2_: Peak oxygen consumption; VE/VCO_2_ slope: Minute ventilation–carbon dioxide production relationship; GET: Gas exchange threshold; COP: Cardiorespiratory optimal point; PetCO_2_: Partial pressure of end-tidal carbon dioxide.

**Table 5:** Evaluation of traditional and alternative thresholds cut-off values of the composite endpoint.

	Female (*n* = 95)			Male (*n* = 363)		
Exercise Testing Parameters	Specificity	Sensitivity	Youden (*J*) Index	Specificity	Sensitivity	Youden (*J*) Index
pVO_2_ ≤ 12 mL/kg/min *	94%	40%	0.34	91%	21%	0.12
pVO_2_ ≤ 14 mL/kg/min	**80%**	**80%**	**0.60**	82%	47%	0.29
pVO_2_ ≤ 15 mL/kg/min	67%	80%	0.47	**79%**	**57%**	**0.36**
VE/VCO_2_ slope > 35	**75%**	**90%**	**0.65**	66%	57%	0.23
VE/VCO_2_ slope > 32	68%	90%	0.58	**57%**	**78%**	**0.35**
Percent of predicted pVO_2_ ≤ 50%	89%	60%	0.49	78%	48%	0.26
Percent of predicted pVO_2_ ≤ 55%	**86%**	**90%**	**0.76**	69%	60%	0.29
Percent of predicted pVO_2_ ≤ 58%	81%	90%	0.71	**63%**	**69%**	**0.32**

* pVO_2_ ≤ 12 mL/kg/min (≤14 if the patient is intolerant to β-blockers). The highest Youden index (*J*) of each CPET variable is highlighted in bold. pVO_2_: Peak O_2_ consumption; VE/VCO_2_ slope: Minute ventilation–CO_2_ production relationship.

## Data Availability

The data presented in this study are available upon request from the corresponding author. The data are not publicly available due to patient consent regarding availability of individual patient data, applicable only to the local investigation team.

## Supplementary Materials

The following supporting information can be downloaded at: https://www.mdpi.com/article/10.3390/life13101985/s1, Figure S1: ROC curves for the composite endpoint in a 36-month follow up. (a) Percent of predicted peak oxygen consumption (pVO_2_) in female patients. (b) Percent of predicted pVO_2_ in male patients; Figure S2: Survival analysis for the composite endpoint in female patients and male patients according to (a) the International Society for Heart and Lung Transplantation (ISHLT) thresholds of percent of predicted peak O_2_ consumption (pVO_2_) ≤ 50% and (b) thresholds of percent of predicted pVO_2_ ≤ 55% in females and percent of predicted pVO_2_ ≤ 58% in males.
