# Supplementary material for: Cardiopulmonary Exercise Testing in Patients with Heart Failure: Impact of Gender in Predictive Value for Heart Transplantation Listing
Source: Life (Basel). 2023 Sep 29;13(10):1985. doi: 10.3390/life13101985 (PMC10608092; doi:10.3390/life13101985)
Supplement: Supplementary file 1 [file life-13-01985-s001.zip › life-2603640-supplementary.pdf]

36-month follow-up

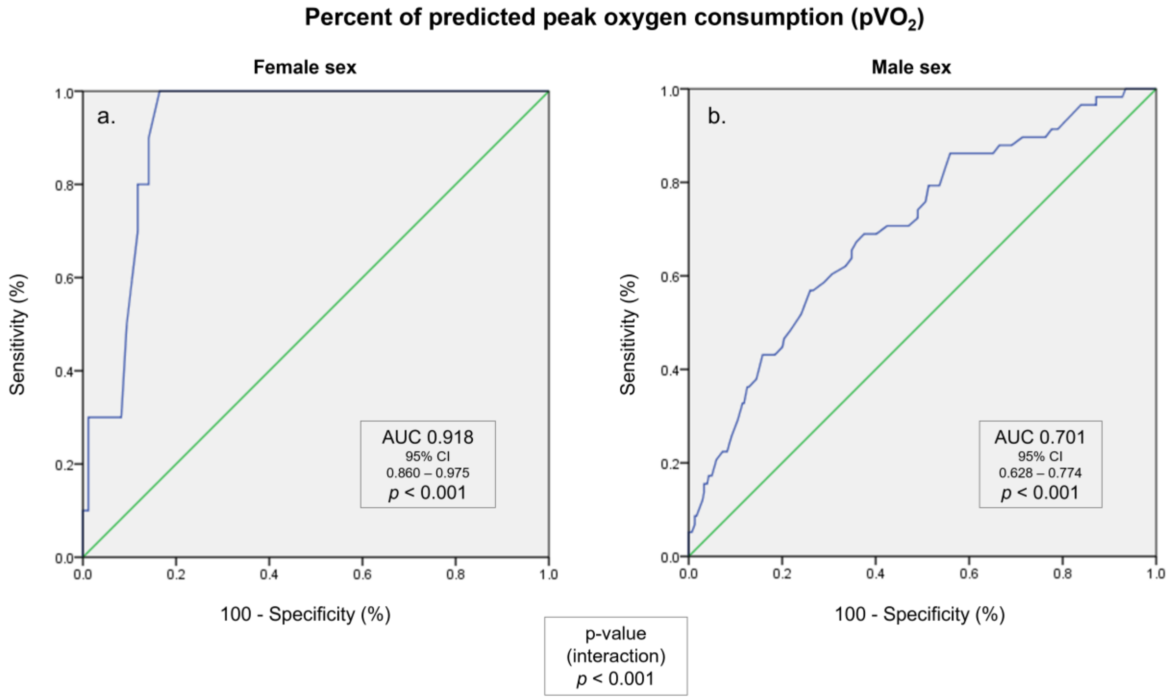

Figure S1: ROC curves for the composite endpoint in a 36-month follow up. (a) Percent of predicted peak oxygen consumption (pVO_2) in female patients. (b) Percent of predicted pVO_2 in male patients.

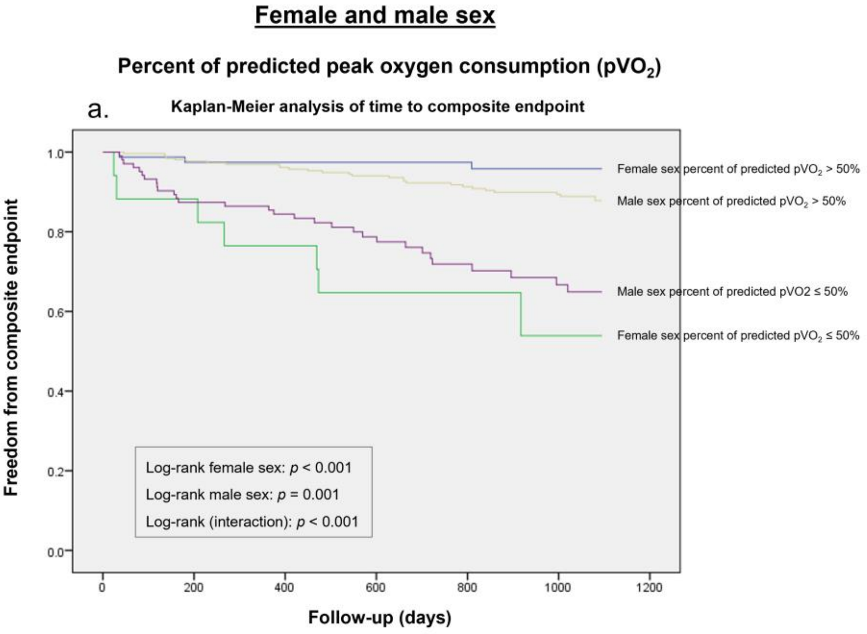

No. at risk							
Female sex percent of predicted pVO$_2$ ≤ 50%	17	15	13	11	11	10	10
Female sex percent of predicted pVO$_2$ > 50%	78	76	76	76	76	75	75
Male sex percent of predicted pVO$_2$ ≤ 50%	103	90	87	82	77	74	73
Male sex percent of predicted pVO$_2$ > 50%	260	254	250	245	239	235	232

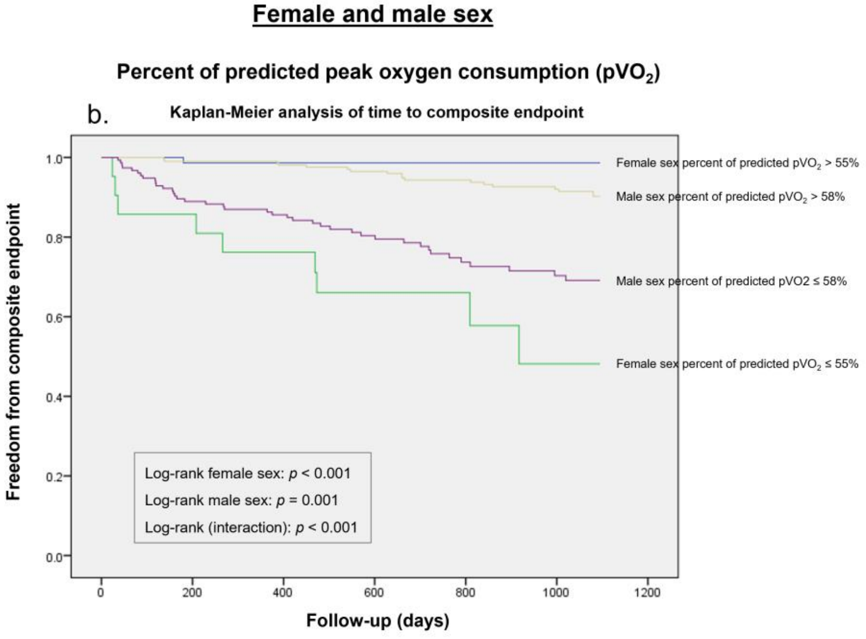

No. at risk							
Female sex percent of predicted pVO$_2$ ≤ 55%	21	18	16	14	14	12	12
Female sex percent of predicted pVO$_2$ > 55%	74	73	73	73	73	73	73
Male sex percent of predicted pVO$_2$ ≤ 58%	154	137	132	125	118	115	114
Male sex percent of predicted pVO$_2$ > 58%	209	207	205	202	198	194	191

Figure S2: Survival analysis for the composite endpoint in female patients and male patients according to (a) the International Society for Heart and Lung Transplantation (ISHLT) thresholds of percent of predicted peak O_2 consumption (pVO_2) ≤ 50% and (b) thresholds of percent of predicted pVO_2 ≤ 55% in females and percent of predicted pVO_2 ≤ 58% in males.
